# The Progress of Multi-Omics Technologies: Determining Function in Lactic Acid Bacteria Using a Systems Level Approach

**DOI:** 10.3389/fmicb.2019.03084

**Published:** 2020-01-28

**Authors:** Shane Thomas O’Donnell, R. Paul Ross, Catherine Stanton

**Affiliations:** ^1^Teagasc Food Research Centre, Moorepark, Fermoy, Ireland; ^2^Department of Microbiology, University College Cork – National University of Ireland, Cork, Ireland; ^3^APC Microbiome Ireland, Cork, Ireland

**Keywords:** lactic acid bacteria, multi-omics, microbiome, genomics, transcriptomics, proteomics, metabolomics, meta-omics

## Abstract

Lactic Acid Bacteria (LAB) have long been recognized as having a significant impact ranging from commercial to health domains. A vast amount of research has been carried out on these microbes, deciphering many of the pathways and components responsible for these desirable effects. However, a large proportion of this functional information has been derived from a reductionist approach working with pure culture strains. This provides limited insight into understanding the impact of LAB within intricate systems such as the gut microbiome or multi strain starter cultures. Whole genome sequencing of strains and shotgun metagenomics of entire systems are powerful techniques that are currently widely used to decipher function in microbes, but they also have their limitations. An available genome or metagenome can provide an image of what a strain or microbiome, respectively, is potentially capable of and the functions that they may carry out. A top-down, multi-omics approach has the power to resolve the functional potential of an ecosystem into an image of what is being expressed, translated and produced. With this image, it is possible to see the real functions that members of a system are performing and allow more accurate and impactful predictions of the effects of these microorganisms. This review will discuss how technological advances have the potential to increase the yield of information from genomics, transcriptomics, proteomics and metabolomics. The potential for integrated omics to resolve the role of LAB in complex systems will also be assessed. Finally, the current software approaches for managing these omics data sets will be discussed.

## Introduction

Sequencing the first whole genome of a bacterial strain, namely *Haemophilus influenzae*, in 1995 was a milestone in molecular biology for a number of reasons (Fleischmann et al., 1995), one of which was heralding in an era rich in information where the volume of data produced was beyond being completely interpreted. Subsequent genomic data sets derived from bacteria elucidated gene functions, metabolic networks, biological pathways, microbial evolution and genome structure significantly enhancing our understanding of bacterial function and potential (Kanehisa and Goto, 2000; Ley et al., 2006). However, the challenge in deciphering relevant genetic information from the background genetic material was almost an impossible task. To combat this, more information was required. Information on the transcription of the genetic material and subsequent production of proteins was necessary. The targets of these proteins and the molecules they interact with had to be determined. Nuanced epigenetic triggers and the metabolites that are ultimately produced are all crucial to understanding the system as a whole. Indeed, much of the observed phenotype in a system can be explained in the context of these data sets when interpreted correctly.

Biological systems rely on the DNA – RNA – protein information transfer paradigm that determines the phenotype of an organism. Biologists have analyzed these “omes” for a number of years in the form of genomics, transcriptomics and proteomics. In addition to these, epigenomics and metabolomics have recently been used to answer specific questions relating to the many functions of an organism. Given the year on year advances in “omics” technologies, the volume of information that can be gathered in individual studies is expanding rapidly. Furthermore, the current high throughput nature of these techniques has increased accessibility to this information in terms of time and cost. This has placed many researchers in a situation where they can collect several omics data sets on the same experimental samples. In order to draw more comprehensive conclusions on biological processes these data sets must be integrated and analyzed as a holistic system.

Technologies involved in a multi-omics approach share several commonalities, some of which contrast with the original approach of molecular biologists. For the most part, molecular biology has utilized a reductionist approach to date. This methodology involves breaking a complex problem into its constituent parts and solving them individually. While reductionism has had significant successes, particularly when the experimental subject is controlled by a single component (Isberg and Falkow, 1985), or can be explained by interactions between single molecules (Krebs, 1940), it also has substantial limitations. These limitations are caused by the process of isolating components of a complex system; often the nature of their role in the system is lost. This is the most significant advantage that omics technologies can have compared to a reductionist approach. Maintaining these components in the system allows observation in a realistic environment where emergent properties can also be studied. These high throughput, top down methods also provide a phenomenal volume of data in comparison to the reductionist approach. For example, as much as six terabytes of information can be generated by processing samples in tandem on an Illumina NovaSeq 6000. Finally, omics technologies require significant computational infrastructure in the form of novel algorithms and software to process and analyze the information produced (Berger et al., 2013).

This has placed researchers in a situation where they must adapt to maximize the results from biological data. Biologists must acquire skills in managing and manipulating these vast quantities of data to resolve experimental questions outside the scope of the lab bench. An appreciation of the strengths and limitations of many of these technologies will allow researchers to answer more complex questions and generate more general conclusions. This is a constant arms race as the technologies that underpin the generation of these omic and meta’omic data sets are constantly advancing. The scope of analysis ranges from entire community samples of 10^12^ microbes to exploring the components of single cells. Higher throughput machines are facilitating deeper sequencing than ever before. This sequencing depth is opening new potential use cases such as metatranscriptomics of the gut microbiome. Integrating these large data sets to provide a systems level view will reveal previously unattainable information on the individual microbes involved.

LAB are uniquely placed to take full advantage of these fundamental changes in approach. Notably, LAB have been the subject of extensive research exploring specific attributes and functions of isolated cultures (Noike et al., 2002; Leroy and De Vuyst, 2004). In depth analysis of individual LAB strains has provided a wealth of information on their biological processes and functionality in a variety of publically available databases. Multi-omics technologies stand ready to exploit this information on LAB to decipher and predict many functions of interest using a systems level approach. Inter-microbe interactions, particularly within starter cultures, are some of the first to witness the potential in these advances (Sattin et al., 2016; Sirén et al., 2019). Similarly, multi-omics technologies are currently being used to tackle more complex systems such as the gut microbiome and host-microbe interactions (Turroni et al., 2016; Huang et al., 2017; Wang et al., 2019). This emerging field is capable of providing a platform for more accurate functional prediction of LAB in a variety of complex environments.

In this review, we will discuss the current most popular combinations of different omics technologies to facilitate accurate functional prediction for LAB. The most recent advances in the relevant technologies will be mentioned, while the potential they hold for deciphering the final phenotype of these microbes will be assessed. Finally, the computational barriers associated with integrating complex and diverse data sets will be discussed.

## The Potential for Functional Prediction in Lab Using Omics Technologies

LAB are among the most industrially significant groups of bacteria. These versatile microbes have a variety of potential functions that are applied in many sectors. Food production, health promotion, production of antimicrobials and *in vivo* fermentation all see benefit from this group of microorganisms. These diverse functions encoded within single genomes are a source of valuable information available for exploitation. As a result, these processes have been studied extensively using *in vitro*, *in vivo* and more recently *in silico* techniques to determine the critical pathways underpinning the phenotypes. Analysis of these molecular pathways has resulted in more accurate use of LAB in commercial endeavors such as starter fermentation cultures and probiotic supplements (Leroy and De Vuyst, 2004; Muñoz-Atienza et al., 2013). Deciphering the underlying biological attributes associated with these critical microbes allows greater understanding of their current roles and may reveal new applications. However, this scrutiny has often focused on a single element of interest in isolation, instead of taking the entire system into account.

A more inclusive approach is possible with diverse sets of information interrogated by omics technologies. To date, many groups have utilized both omics and multi-omics data sets to advance this field and progress knowledge of LAB function (Lahtvee et al., 2011; Rebollar et al., 2016; Huang et al., 2017; Filannino et al., 2018; Ellepola et al., 2019). The consistent progression of new technologies and methodologies has resulted in many studies providing crucial information on the function of LAB. Progress in this manner also provides direction for future work with these microbes. Studies using multi omics for mammalian or bacterial cells may pre-empt similar work with LAB. New methodologies incorporating omics technologies are often applicable to a variety of research topics. These studies will be discussed to gain insight into the potential use cases for LAB. Translating the relevant findings in multi omics research will focus on what can be discovered in LAB within ecosystems they inhabit and their common commercial applications. The influence exerted by the new technological advancements will also be assessed for their direct effect on LAB.

Basic functions of LAB are well understood, as are the cellular processes underlying them. With this foundation of knowledge, research into LAB is in a prime position to exploit the coming wave of omics technologies. The sections below document the strengths and limitations of each omics technology, the impact of each omic data set on inferring LAB function to date, the relevant advancement in technology and how these new methodologies may accelerate future progress.

## The Impact of Genomics on Lactic Acid Bacteria

Genomic information has recently become essential when studying microbes in detail. These data sets can provide an immutable link to the organism that for the most part remains constant. In well studied bacteria, such as many LAB, fully sequenced genomes are readily available in a variety of species (Chenoll et al., 2015; Inglin et al., 2018). These may be used as reference genomes when assembling draft genomes of the strain at hand. Mining these genomes alone reveals information on all traits available to the microbe. Potential products are inferred from the genetic code and viable pathways can be determined analyzing the relevant genes. These pathways are categorized by their overarching function e.g., carbohydrate utilization pathways. Comparative genomic analysis between the generic pathways available to LAB and the strain of interest may highlight the unique functional capacity of the particular strain of interest (Makarova et al., 2006). *De novo* construction of genomic information is also possible. Knowledge of characteristic motifs and patterns in specific alignment of DNA bases e.g., Shine-Dalgarno, allows software to detect important genes such as cluster specific transcription factors and the promoters associated with said genes (Wolf et al., 2018). Such methods can be used in conjunction with comparative genomics to predict the production of difficult to detect molecules such as secondary metabolites in the form of antibiotics, toxins and immunosuppressives (Zerikly and Challis, 2009; Weber et al., 2018). Mining genomes for well-known genes is more straightforward using BLAST or DIAMOND (Altschul et al., 1990; Buchfink et al., 2014). Searching the DNA or protein sequence of the element of interest against your genome will provide probability-based results on its presence in the genome. This information is the bedrock on which the multi-omics integration is built.

Mining genomic information on its own may direct experimentation or suggest mechanisms for known functional attributes. This was exemplified by analysis of genomic data in *Lactobacillus ruminis* revealing the presence of functional flagellar apparatus in the form of 45 flagellar genes (Neville et al., 2012). Robust flagellar apparatus suggests *L. ruminis* is a motile microbe and presents a mechanism for pro-inflammatory tendencies. Despite not expressing flagella in culture media, strains with the genomic capacity to produce flagella were observed to partially recover this ability *in vivo*. Gene clusters for crucial mucus binding pili have been detected in several LAB (Douillard et al., 2013). This gene cluster explains *L. rhamnosus*’ capacity to adhere to the intestinal mucosa (Kankainen et al., 2009). In a similar fashion, gene clusters that are capable of producing a broad range of bacteriocins have been reported. This information led to observe *Lactobacillus salivarius* outcompeting *Listeria monocytogenes* utilizing these compounds (Corr et al., 2007).

Appropriate use of secondary metabolite software tools for analyzing the genome has resulted in the discovery of many novel antibiotics (Schulze et al., 2015; Tian et al., 2016). Incorporating software such as anti-SMASH (Blin et al., 2017), PRISM (Skinnider et al., 2017) and GRAPE (Dejong et al., 2016) has facilitated mining of the genomic data sets for crucial biosynthetic gene clusters. A very similar process unlocks the potential in genomic data sets of LAB. These microbes are capable of producing a wide variety of diverse anti-microbial peptides (Stoyanova et al., 2012; Zacharof and Lovitt, 2012). Capitalizing on these powerful analysis tools can realize much of the potential that a genomic data set provides and determine many possible functions available to the bacterium in question. Researchers can forgo the culture based issues with screening for novel anti-microbial compounds and instead direct future experiments more accurately. This process was adeptly demonstrated by Singh S. et al. (2015), to identify putative bacteriocins (Singh N.P. et al., 2015). Twenty LAB genomes were assessed for relevant bacteriocin producing genes. Putative operons were identified leading to further characterization of novel bacteriocins. This simplistic process describes the exploitation of genomic material to identify these traits of interest.

The utility of genomic information is on the cusp of a generational leap forward. Third generation sequencers, such as the Sequel II and the MinIon, are set to remedy many of the intrinsic issues associated with second generation sequencers (Rhoads and Au, 2015; Lu et al., 2016). These are primarily a GC bias in fragmentation and amplification (Grokhovsky et al., 2011; Poptsova et al., 2014), short reads resulting in difficult to sequence repeat regions (Bovee et al., 2007; Aird et al., 2011; Genovese et al., 2013) and substantial burdens on computational rearrangement of genomes from a huge volume of short reads (Aldrup-Macdonald and Sullivan, 2014; Sims et al., 2014). Limitations associated with short read sequencing become apparent when analyzing large insertion sequences (Iranzo et al., 2014) or substantial rearrangements in chromosomal or circular genomic sequences (Darling et al., 2008; Sobreira et al., 2011). Transposable elements often contain genes of interest encoding traits such as antibiotic resistance and bacteriocin production (Klaenhammer, 1993; Toomey et al., 2009). In some cases, critical processes such as genome replication are also intrinsically linked to the presence of inverted repeats (El Kafsi et al., 2017) and structure variants of large genomic transfers between species often harbor crucial mechanisms (Kant et al., 2011). Progress in this area will have a significant impact on determining some of the poorly understood traits associated with LAB (Teusink and Molenaar, 2017). Similarly, it will be possible to shed more light on up-and-coming areas for LAB such as discovering novel bacteriocins (Perez et al., 2014). The ability to analyse far greater read lengths (>20 kb) has facilitated the characterization of clusters coding resistance to crucial antibiotics as well as accurate tracking of large translocations of patho-adaptive traits from commensals to pathogenic bacteria in the microbiota (Huang et al., 2016; Proença et al., 2017). LAB are likely to have gained traits from similar translocation events.

However, this is the proverbial tip of the iceberg regarding the potential impact on LAB due to third generation sequencing. The most striking example of this is the extraordinary amount of information in the form of epigenetics that is lost due to the fragmentation and sequencing process in next generation sequencing. Epigenetic, post transcriptional modifications exert significant control on bacterial genomes resulting in altered phenotypes (Goldberg et al., 2007). Currently, there is a significant cost in both time and money associated with determining epigenetic marks (Kurdyukov and Bullock, 2016; Soto et al., 2016). Both Nanopore and PacBio report the presence of epigenetic modifications during sequencing (van Dijk et al., 2018). This will result in regular sequencing reporting epigenetic alterations to bases, in turn opening this added layer of complexity to a far greater audience (Casadesús and Low, 2006).

## Overview of Omics Technologies

The following section will review the existing omics technologies in the context of LAB. The advantages and disadvantages of each is summarized in Table 1.

**TABLE 1:** Strengths and weaknesses of the individual omics technologies described in this review.

**Data set**	**Strengths**	**Limitations**	**Recent advances**	**Citations**
Genomic	Immutable link to the organism; databases of reference genomes often available to aid reconstruction; provides a static image of genes of interest; high throughput sequencing as standard	Short read sequencing results in gaps in “hard to sequence” regions; impossible to determine the activity of the genetic elements sequenced; difficult reconstruction of genomes with bioinformatic software	Third generation sequencing; simultaneous epigenetic determination with genome sequencing; higher throughput for shotgun meta-genomics	Bovee et al., 2007; Sims et al., 2014; van Dijk et al., 2018
Transcriptomic	Robust data on the requirements of a microbe in a given environment; vast quantity of data is produced; effective combination with single cell technologies	RNA isolation and sequencing are susceptible to handling errors; the transient nature of RNA only provides a snapshot of the needs of the organisms; presence of RNA’s does not necessarily predict the translation into proteins	Higher throughput Next Gen Sequencers (NovaSeq 6000); meta-transcriptomics of large systems is now possible; more reliable software for integration and variant determination	Lohse et al., 2012; Vogel and Marcotte, 2012; Liu et al., 2016; Dagogo-Jack and Shaw, 2018
Proteomic	Significant database of known proteins provide a strong platform to predict function; robust link between an organisms proteomic profile and its phenotype; provides a more stable image of the current requirements of the organism than other omics technologies	Throughput capabilities of proteomics lags behind other omics; expensive MS machinery is required for proteomic research; concessions are made in order to analyze the vast array of proteins – Splitting large proteins into smaller sections to facilitate MS analysis	Orbitrap Mass Spec facilitates ionization of more complex proteins; combining liquid chromotography with multiple MS’s allows accurate depiction of specific groups of proteins; powerful analytical tools i.e., PECAN, facilitate more accurate predictions from untargeted proteomics	Pascal et al., 2008; Michalski et al., 2012; The Uniprot Consortium, 2014; Ting et al., 2017; Monaci et al., 2018
Metabolomic	Direct connection between phenotype and metabolomics profile; provides an image of many well-studied metabolites simultaneously; diverse range of applications across many fields	The transient nature of metabolites makes them susceptible to sampling artifacts; numerous costly LC/GC and MS machines needed for processing	Back to back LC or MS machines provide higher resolution of specific groups e.g., LC-MS/MS; new machinery such as High Temperature-Ultra High Performance LC are overcoming previously difficult to detect metabolites; single cell sorting advances are facilitating robust single-cell metabolomics in the near future	Wang et al., 2014; Chetwynd et al., 2015; Rebollar et al., 2016; Ferrocino and Cocolin, 2017; Zhang and Vertes, 2018

*The recent advances in their respective fields are included to illustrate the frequent progress in this area. As discussed below, many of the weaknesses that are mentioned may be overcome by integration of several of these omics technologies together.*

### Single Cell Genomics

Single cell sequencing approaches have become more frequently utilized throughout the past decade (Tang et al., 2009). This approach holds significant promise for several reasons, primarily due to its potential to decipher cellular differences within heterogenous cell populations in any tissue or cell culture. Determining cell heterogeneity is an essential step in understanding the development, regulation and response to external influence in a population of cells. This natural heterogeneity is amassed and averaged in bulk sequencing approaches. Traditional sequencing removes much information that may indicate more nuanced reasons for phenotypes of interest. Many techniques have been developed to isolate and sequence these single cells in a cost effective and high throughput manner (Wang and Navin, 2015; Lan et al., 2017; Hwang et al., 2018). Microfluidics and Fluorescent Activated Cell Sorting (FACS) are the most popular methods to date. FACS relies on tagging and isolating fluorescent cells by capitalizing on the charged nature of a fluorescently tagged cell (Gross et al., 2015). Microfluidics focuses on the precise combinations of oil, surfactants and cells to create a droplet containing a single cell (Lecault et al., 2012). These techniques are used in a variety of fields and are perfectly designed for use in single cell sequencing. Furthermore, these techniques are adapted to include lysing of cells and to incorporate sequencing materials within the droplets encapsulating the cell components.

Single cell sequencing technologies have focused on human cells to date. This is in part due to the ease involved in lysing them to release nucleic acids, enabling high throughput protocols. This issue is being addressed to link higher throughput cell isolation methods, such as microfluidics, including suitable lysing protocol for bacterial cells (Liu et al., 2018). For this reason, however, LAB studies availing of high throughput analysis are not presently available. Despite this, proof of principle studies demonstrate the potential for LAB research in this area. Large sequencing attempts to explore the “microbial dark matter” of unknown areas of the tree of life (Rinke et al., 2013) in microbiome samples have been conducted. In a similar manner, single cell isolation techniques may also be employed to analyze the least abundant bacterial species within community samples. Minor community members have been observed within the fermented dairy product Koumiss using this approach (Yao et al., 2017). The protocol, described by Yao et al., 2017, involves diluting microbiome samples and sequencing single cells. This powerful, yet simplistic, technique can be exploited to analyze pools of bacteria that are known to have a specific output or phenotype in order to isolate the cells responsible. Analyzing minute quantities of DNA and RNA, sometimes as low as femtograms of material, are within the remit of these single cell techniques (Lasken, 2007). This knowledge has been utilized in environmental samples to isolate microbes of interest such as oil degrading microorganisms (Mason et al., 2012). Furthermore, single cell segmented filamentous bacteria were isolated using microfluidics from mouse gut microbiome samples (Pamp et al., 2012). This protocol provides an isolation method applicable for single cell LAB in community samples. Despite the lack of single cell sequencing studies on LAB, many of the techniques described are directly applicable to LAB. These highlight the potential advances that are attainable in this area. The rapid progress of isolation technologies, lysing protocols and sequencing depth will provide a more stable platform for targeted single cell analysis of LAB (Gawad et al., 2016).

### Metagenomics

In contrast to single cell genomics, metagenomics provides community-based genome sequences of many diverse species simultaneously. This information allows correlation-based work to compare the abundance of particular gene families to the respective environment (Brown et al., 2011). Metagenomics provides an overview of species abundance in the microbiome and characterizes common metabolic pathways available in the ecosystem (Huttenhower et al., 2012). The contribution LAB make to the gene pool and functional processes can be discerned using metagenomic data. Armed with this knowledge, the potential role LAB play in the community can be determined. Metagenomics is regularly used to determine the microbial diversity in order to direct further analysis of the sample at hand. Zhang et al., 2016, used metagenome sequencing to study novel fermented foods. These insights into fermented foods revealed potentially interesting *Lactobacillus* strains that were then isolated from the samples (Zhang et al., 2016). This targeted approach to omics data is the most effective method when working with a single omics set. However, combining other omics data unveils a more dynamic image of the metagenome. This is most commonly applied when integrating genomic and transcriptomic data. These data can be indispensable to understanding the functional role members fill in a given system.

## Combining Transcriptomics With Genomic Data Sets

The combination of genomics and transcriptomics is one of the most common in addressing experimental questions. Combining transcript data with available genomic information provides an image of the intentions of the organisms, given a specific environmental situation. The integration of genomic and transcriptomic data (Curtis et al., 2012; Ju et al., 2012; Craig et al., 2013; Lappalainen et al., 2013) is frequently used across many fields, while merging metagenome and metatranscriptome data (Shi et al., 2010; Solbiati and Frias-Lopez, 2018) is becoming far more prevalent. Genomic and transcriptomic data sets have been combined regularly to offer insight into the role LAB play in food spoilage (Andreevskaya et al., 2015), potentially probiotic traits of LAB strains (Saulnier et al., 2011) and their ability to use alternative electron acceptors in order to respire instead of ferment (Brooijmans et al., 2009). Isolating strains with particular functions is readily facilitated by transcriptomics data. A systems approach was used to analyze the altered functional capacity of a mutated *Lactococcus lactis* strain utilizing genomic and microarray data (Chen et al., 2015). The genetic component responsible for its increased thermo resistance was determined using this combination of omics data. Transcriptome analysis of *Lactobacillus* strains causing beer spoilage was performed to determine the functional pathways which enable these microbes to enter the viable putative non-culturable (VPNC) state and thus survive in beer. Analysis of three *Lactobacillus acetotolerans* strains revealed that these strains were in a heightened stress state and had reduced gene expression levels in several other regular pathways such as metabolic processes, transport and enzyme activity. Understanding this process may afford future opportunities to prevent beer spoilage by inhibiting entry into the VPNC state (Liu et al., 2016).

Fundamental processes in LAB such as amino acid and carbohydrate metabolism have been advanced using transcriptomic data. Comparative transcriptomics has been imperative in understanding amino acid metabolism in *Lactococcus lactis* MG1363. A codY mutant strain was used to determine the role this gene plays in regulating more than 30 genes involved in metabolizing amino acids (den Hengst et al., 2005; Guédon et al., 2005). This strain was further analyzed using transcriptomic data to analyze its global regulatory networks during growth in milk (de Jong et al., 2013). Knowledge regarding the expression of critical genetic components in LAB such as the catabolite control protein A (CcpA) has seen considerable advancement using transcript data. Deep transcriptomic and physiological data were used to explore the differential expression between WT *Lactobacillus plantarum* and a CcpA mutant during growth phase on different carbohydrates (Lu et al., 2018). This study reports a substantial rearrangement in the carbohydrate metabolism regulatory network and sheds new light on the complexity of this process. It is clear that incorporating this data set into LAB research has already led to an increased understanding of these microbes.

### Single Cell Transcriptomics

Transcriptomics at a single cell resolution is a relatively new field that may unravel many of the changes in transcription that are altered through a cells life span. These alterations are completely masked by bulk transcriptomics (Shapiro et al., 2013). With new technologies providing faster delineation of single cells (Klein et al., 2015) in conjunction with greater sequencing depth with machines such as the NovaSeq, large scale single cell transcriptomics is more accessible than ever. Progress in these complimentary fields allows researchers to explore a previously unavailable aspect of cell state heterogeneity. Many single cell transcriptomic studies focused on stem cells (Kolodziejczyk et al., 2015), embryos (Yan et al., 2013), tumors (Patel et al., 2014) and the nervous system (Zeisel et al., 2015). The use cases for this approach in these tissue types are apparent due to the advantages of delineating the differences between differentiated and non-differentiated cells. Comparing the responders to non-responders provides a greater opportunity to isolate mechanisms that stimulate desired responses (Hidalgo-Cantabrana et al., 2012; Shalek et al., 2013). This method is also efficacious when the genome and transcriptome sequencing are carried out simultaneously on the same cell (Macaulay et al., 2015). These researchers sequenced the DNA and RNA of single mammalian cells in parallel, demonstrating the current capacity of single cell technologies. A subpopulation of 10% within 172 single cells of human and murine origin was reported after analysis. Several genetic alterations between cells and large chromosomal translocations events were observed.

LAB are among the best understood constituents within the microbiome. However, it is difficult to determine the importance of their role in an ecosystem this large without a systems approach (Pessione, 2012; Waldor et al., 2015). Constituents of the microbiome are known for their ability to affect the impact of drug compounds and therapy (Lindenbaum et al., 1981), ferment and convert many components in our diet (Albenberg and Wu, 2014) and have significant impact on healthy brain function (Foster and McVey Neufeld, 2013). Single cell technologies may lead the way in deciphering LABs role in these functions of the microbiome.

### Metatranscriptomics

Advances in next generation sequencing technology have reached a sequencing depth that facilitates more comprehensive metatranscriptomics of large community samples such as the gut microbiome (Bashiardes et al., 2016; Furnholm et al., 2017; Mehta et al., 2018). The current NovaSeq can produce 20 billion reads in a machine run. With this volume of reads between 100–400 taxa can reach maximum saturation of reads required for the highest statistical power (Ching et al., 2014). This step forward provides a powerful tool for gut microbiota analysis and realizes a true systems biology approach to determining how this community reacts to environmental perturbations. Due to the large database of sequenced LAB genomes, LAB stand to gain the most from the analysis of ecosystems such as the gut microbiome with a systems level approach. This combination of metagenomics and metatranscriptomic data is just beginning to become relevant in larger ecosystems; however, significant results have already been attained.

To date, this approach has been observed in smaller microbiome samples such as Kimchi (Jung et al., 2013) and rumen (Kamke et al., 2016). This methodology was also used in a proof of principle analysis to determine the function of a critical microbe in bacterial vaginosis. Indeed, by utilizing metatranscriptomics *Lactobacillus iners* was implicated in having a functional role in the presence of this disease differentially expressing over 10% of its genome between healthy and disease states (Macklaim et al., 2013). Specific commercially important processes such as cheese ripening have also seen the impact of this approach. Despite using shallower metatranscriptome data, De Filippis et al., 2016 demonstrated temperature-driven functional changes in the cheese microbiome during ripening which had a significant impact on cheese maturation rate. They indicated that “processing-driven microbiome responses” can be altered to influence product quality and production efficiency (De Filippis et al., 2016). Expression data that can be tracked throughout the process and related back to the specific strains responsible is invaluable in important commercial processes such as cheese production. The potential to carry out similar research in large microbiome samples such as the gut, in a manner similar to De Filippis’ experiment, is fast approaching.

Third generation sequencing platforms may have a direct impact on the RNA-seq field. Long read technologies perform better in the determination of unknown transcript abundance in single celled organisms (Tombácz et al., 2018), full-length splice isoforms with alternative splicing (Xu et al., 2017) and co-transcription of genes in a polycistronic fashion (Tardaguila et al., 2018). It is clear that there are obvious advantages to long read sequencing in reducing the difficult reassembly and loss of contextual information associated with short read sequencing. However, the lower accuracy and sheer scale of some transcriptomics and metatranscriptomics projects keeps them out of reach of current third generation sequencers.

Integrating metatranscriptomics with deep metagenomic data presents the ability to track a time mediated response to specific changes in the environment. Be it antibiotic exposure, pathogenic infection or probiotic administration, a wealth of information on how the system is reacting to the alteration will be generated. Such data could be tracked back to each species and provide information on how to produce effective therapies in similar situations. This holistic approach to studying these bacteria in their natural habitat will reveal much about the production of compounds of interest and may result in interesting revelations about the transition between the microbiomes symbiotic and dysbiotic states.

## Assessing the Utility of Proteomics Within Multi-Omics Data

Proteomics investigates the complete set of proteins present in a cell, tissue or organism at a molecular level. Proteomics in its own right is a powerful analytical tool that has helped resolve many functional questions regarding LAB. Proteomic analysis has determined abundant compounds that are present in the transition between growth phases in LAB (Pessione et al., 2005) and has been used to study the metabolic interactions of LAB (Pessione et al., 2010). Proteomics has determined critical proteins involved in acid stress resistance in *Lactobacillus casei* comparing a known stress resistant mutant to the Wild Type (WT) strain (Wu et al., 2012). Assessing the complete set of secreted proteins in LAB has increased our understanding of how these bacteria interact with their environment (Zhou et al., 2010). Research to determine the capacity for LAB to resist osmotic stress, a critical trait of all microbes in challenging environments, has also progressed notably utilizing proteomics (Zhang et al., 2010). These examples serve to prove the flexibility and effectiveness of proteomics; however, this information is best used when combined with other omics data sets. Despite considerable advances in proteomic technologies of late (Michalski et al., 2012; Hein et al., 2013; Gillet et al., 2016), this omics data set is the limiting factor in relation to throughput when integrated with genomics and transcriptomics. Untargeted discovery proteomics is termed Data Independent Acquisition (DIA) and allows the most comprehensive combination with other omics sets (Hu et al., 2016). This process facilitates the tracking of genes to proteins in a manner that produces functional data (Tocchetti et al., 2015; Trapp et al., 2016; Kedaigle and Fraenkel, 2018).

Protein abundance is intrinsically linked to the mRNA levels discussed above, however, mRNA abundance does not correlate well to protein abundance in a system (Chen et al., 2002; Pascal et al., 2008; Vogel and Marcotte, 2012). Considering this disparity, and that proteins are the molecules that control almost all cellular processes, the benefit from integrating these technologies for a more complete image is clear (Griffin et al., 2002; Cox and Mann, 2007). When combined, these data sets can answer higher dimensional questions about large scale processes such as studying the metabolism of many products simultaneously in a holistic manner (Delmotte et al., 2010; Wang et al., 2013). Complex microbial interactions such as quorum sensing may be deciphered using this approach (Di Cagno et al., 2011). This process was carried out to assess the complex interplay between LAB strains in yogurt fermentations. Transcriptomics and proteomics were combined to understand how the strains present interacted to produce the desirable effects. By-products from a single strain stimulated growth in the co-culture resulting in a reliable yogurt formation (Herve-Jimenez et al., 2009; Sieuwerts et al., 2010). Similarly, this information can be used to further understand specific traits of LAB such as their crucial ability to manage bile stress in the case of probiotic strains of *Lactobacillus* (Koskenniemi et al., 2011). Combining proteomics with genomics and transcriptomics allows more robust biomarkers and treatments to be determined. This is observed in traits of disease phenotypes in humans (Wheelock et al., 2013) or functional processes in bacteria (De Keersmaecker et al., 2006). Understanding the methods that bacteria use to interact with their environment through several omic data sets develops network links between these data sets. Primary processes such as stress responses are frequent targets for a combinatory approach and may reveal critical information that would be lost without a holistic approach (Dressaire et al., 2011).

### Single Cell Proteomics

Single cell analysis also makes an impression on the field of proteomics. Until recently, there were merely proof of principle publications describing the ability to identify minute concentrations of proteins available in a single cell (Jo et al., 2007; Rubakhin and Sweedler, 2007). Mass spectrometry, the primary method for proteomics research, is frequently used when tens of thousands of cells are available from which to extract proteins. However, a single cell has in the region of 1 × 10^5^ protein molecules. With this in mind, it is clear why single cell mass spectrometry (MS) techniques will reveal only the most abundant of proteins present. Despite this, advances in this area have increased the scope of single cell proteomics considerably. Progress within the flow cytometry field has resulted in an increased variety of fluorescent markers (Krutzik and Nolan, 2006). Antibody based immunofluorescence confocal microscopy has been used in human cells to identify >12,000 proteins across multiple cell lines (Thul et al., 2017). Microfluidic image cytometry can now analyze activity of specific protein groups such as kinases (Sun et al., 2010) and using photocleavable DNA barcode-antibodies to quantify various proteins from single cells is also possible (Agasti et al., 2012; Ullal et al., 2014). These techniques have enabled targeted proteomics of single cells, however, to our knowledge no single cell proteomics work has been carried out on LAB. Tracking the mechanisms and rate at which single cells adapt to environmental exposures will help define the specific triggers, systems and pathways involved in these situations. Developing knowledge of these networks while avoiding the clouded nature of bulk analysis is the most effective method to increase the accuracy at which we can predict the function and reactions of these microbes.

### Metaproteomics

Currently, meta-proteomics struggles to stack up to the comprehensive nature of metagenomics and metatranscriptomics. However, a recent study by Ting et al. (2017) depicts the current power of untargeted exploratory proteomics accurately. This group demonstrated that the difference between the more comprehensive nature of Data Independent Acquisition and more accurate Data Dependent Acquisition (DDA) is lessening. After developing a novel library free peptide detection method, PECAN, this group was capable of detecting 12,767 peptides within a sample, 6,221 of which were unique compared to the targeted approach. The untargeted DIA approach detected 83% of the peptides elucidated during the targeted DDA approach. The detection accuracy was impressive as ∼99.5% of the retention times were identical between both approaches. This is while simultaneously detecting more than twice the number of peptides, indicating its suitability for exploratory proteomics. Techniques such as this are facilitating more realistic, high throughput proteome analysis (Kim et al., 2014). This represents the future of larger scale metaproteomic work; however, it is still in its infancy. For this reason, targeted assessment of protein abundance and identification is more appropriate and useful than untargeted in its current state. As such a targeted approach must often be taken when combining the information with metagenomic and metatranscriptomics data. It is feasible to choose a specific function or set of functions to analyze as part of the system-wide metaomics approach. Important traits associated with functional features of LAB can be interrogated further with proteomics in this manner (Hamon et al., 2011; Perez Montoro et al., 2018). Analyzing functional traits has developed a significant understanding of the proteome of the LAB group to date (De Angelis et al., 2016). Several studies in 2019 have combined metaproteomic data sets with the genomic counterparts to analyze fermented foods. This phenomenon has revealed much information regarding the central role played by LAB in these functional foods (Xie et al., 2019a, b). However, these metaproteomic approaches may only assess smaller scale community samples. Expanding this process to incorporate gut microbiome samples is beyond current proteomic technologies (Verberkmoes et al., 2009).

## Completing the Multi-Omics Picture: Metabolomics

Metabolomics is the study of all metabolites produced in a given system. This omics technology is a natural progression from proteomics in that proteins are responsible for the presence of the majority of metabolites found in an organism. The far-reaching effect that metabolomics research may have is apparent, as all phenotypes are intrinsically linked to the metabolites involved in the system studied. This concrete connection between the phenotype and metabolome makes it an exciting data set to add to any research. The volume of information that can be gathered is substantial considering the sheer scale of all metabolites that may be present. Recent advances have resulted in more accurate systems than ever for delineating these compounds. Back to back Liquid Chromotography or Mass Spectrometry units (LC/LC-MS, LC-MS/MS) (Cui et al., 2018), High Temperature Ultra High Performance Liquid Chromotography (HT-UHPLC) (Yoshida et al., 2007; Sarrut et al., 2014) and nanoflowUHPLC-nanoESI-MS (Chetwynd et al., 2015) are filling in the gaps for previously difficult to detect compounds such as hydrophilic or minor metabolites. This progress results in untargeted metabolomics reaching the throughput necessary to combine them comprehensively with other omics data sets.

The utility of metabolomics technology on its own is obvious and has been used to make connections between gut related diseases and metabolites produced by gut microbes (Bingham, 1999; van Nuenen et al., 2004; Nicholson et al., 2005). Metabolomics has also been utilized to decipher functions of LAB such as their role in commercially important processes and revealing information regarding their metabolism (Weckx et al., 2010; Wilson et al., 2012; Mozzi et al., 2013). In a study by Hong et al., 2011, probiotic LAB were introduced to a group of Irritable Bowel Syndrome (IBS) sufferers. NMR was used to determine the metabolic niche that these LAB fulfilled in the IBS sufferers by comparing them to a control group not receiving the probiotic (Hong et al., 2011). The resulting data suggested a dysregulation in energy homeostasis and liver function based on the metabolites present. The potential of metabolomic data sets are unlocked when linked to other components throughout the organism such as proteins, RNA etc.

Many functions have been determined integrating these data sets as they provide a powerful platform to answer many research questions. This has been observed in several fields such as chemotherapy toxicity, toxicology, fungal phytopathology and heavy metal resistance in plants (Heijne et al., 2005; Tan et al., 2009; Singh S. et al., 2015; Wilmes et al., 2015). In LAB, this combination of technologies is useful when analyzing an organism-wide response such as growth efficiency and amino acid metabolism (Lahtvee et al., 2011) or the ability to adapt to ecological niches (Heinl and Grabherr, 2017). Conversely, these omics can be used with a specific end point in mind such as probiotic potential to treat a specific issue, providing a powerful protocol for selecting suitable candidates (Rebollar et al., 2016). A combination of genomics, transcriptomics and metabolomics has been used to analyze the microbial role in the fast growing area of milk whey. Milk whey has recently transitioned from a low value by-product to a high value commercial product. However, the regulation has lagged behind resulting in many unknowns regarding the microbial composition and microbial by-products present in this milk whey. Omics data sets allowed Sattin et al., 2016, to report the reasons for poor whey quality, the potentially concerning compounds present and how to maintain higher commercial value (Sattin et al., 2016). Similar processes’ were described when assessing the role multi-omics data may play in food microbial interactions (Sattin et al., 2016; Ferrocino and Cocolin, 2017). Indeed, genomics, transcriptomics and metabolomics aided Turroni et al., 2016, in deciphering the complex interactions that allow *Bifidobacterium* strains to persist in the murine gut (Turroni et al., 2016). This valuable information leads to a greater understanding of the functional requirements for probiotic strains to survive this environment.

**FIGURE 1:**
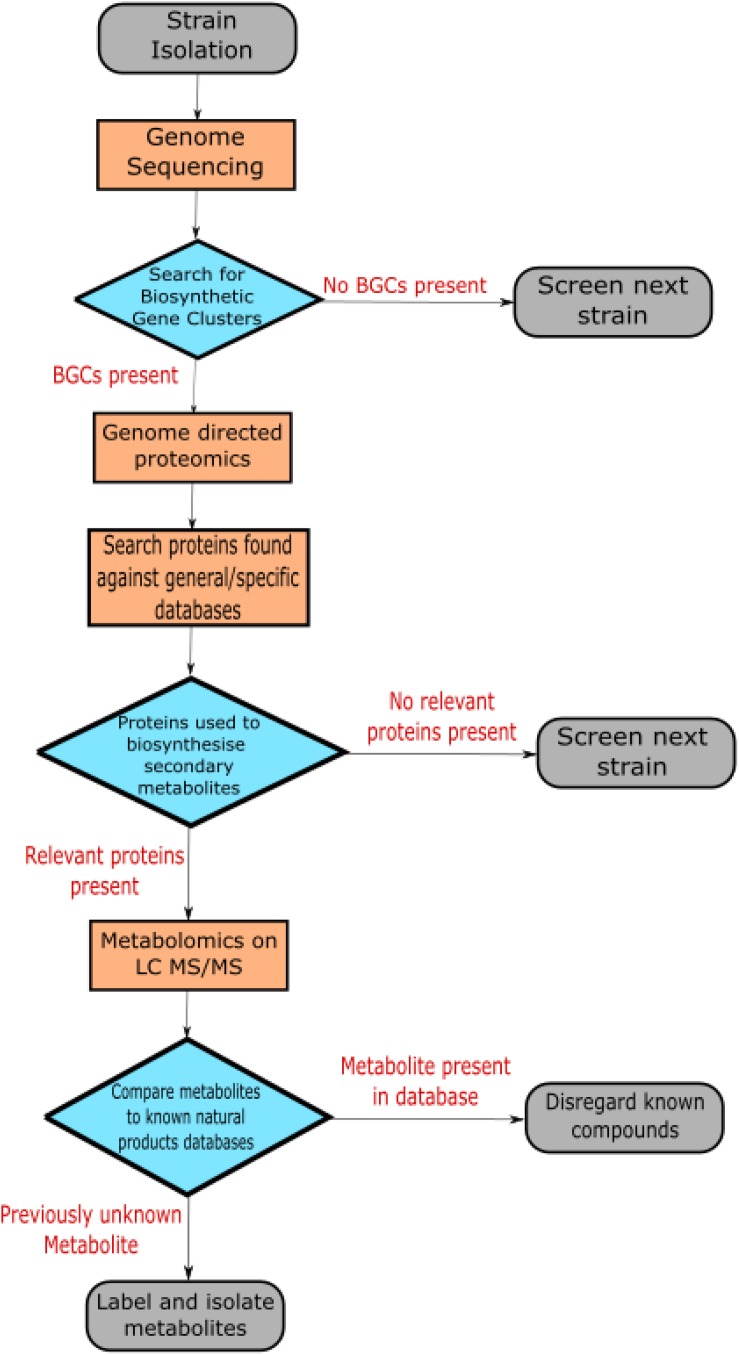
The flow chart depicts a generalized version of the methodology used by Albright et al. (2014) to isolate novel secondary metabolites with antibiotic potential.

Exploiting multi-omics data sets is now a pivotal step in discovering novel antibiotics (Palazzotto and Weber, 2018). Albright et al., 2014, demonstrate a clear methodology for screening strains for novel antibiotic metabolites based on multi-omics data (Albright et al., 2014). Figure 1 shows a generalized version of the method outlined in this paper. The flow begins with sequencing the genome of the strain of interest. This facilitates searching the genome for the presence of biosynthetic gene clusters that may indicate antibiotic production. The genome enabled proteomics used in this study produced >15 fold increase in the number of antibiotic peptides detected compared to regular proteomic analysis. After determining the relevant proteins produced, metabolites were assessed using data-dependant acquisition. Metabolites associated with the proteomic data set that represent potential antibiotic compounds are selected for using LC MS/MS. Isolated metabolites are compared to the relevant databases to rule out the known compounds. In this manner, novel natural products are isolated in an effective progression from strain to isolated compound using multi-omics data.

### Single Cell Metabolomics

Single cell metabolomics, although still in its infancy, can reveal the closest link to the phenotype of a single cell in a population. Sample volumes, low concentrations of analytes and sampling techniques are significant obstacles to this technology based on miniscule substances. However, in recent years, we have seen significant steps in accurate sampling of metabolites. A comprehensive review has been completed by Duncan et al., 2019, on the state of the art techniques developed over the last three years for single cell metabolomics (Duncan et al., 2019). A primary concern of these techniques is the transient nature of metabolites. A cell with particularly fast metabolic turnover can change its metabolic profile in 0.3 s (Zhang and Vertes, 2018). This volatility means the cells must be maintained in the native environment and treated carefully to avoid artifacts of the sampling technique.

Live single cell mass spectrometry shows promise providing rapid, direct analysis of targets while also producing a complete annotation of the results in less than one hour (Fujii et al., 2015). Fluorescent based techniques relying on flow cytometry and direct microscopy are suitable methods for analyzing specific metabolites (Li et al., 2016; Mondal et al., 2017). More recent developments in single cell technologies such as microfluidics are also applicable in isolating cells based on their extracellular metabolite profile (Wang et al., 2014). With associated technologies progressing quickly, it is clear that single cell metabolomics will make large steps with regard to throughput and accuracy in the near future. However, no LAB based single cell metabolomics data are available in published literature. Despite the difficulty in accessing the metabolomic data of single cells, the utility of the information is clear. This analysis allows abundance of specific metabolites to be confidently linked to traits. Moreover, developing specific links will aid functional prediction in all similar microbes with metabolomics data.

The flexibility of these data sets is apparent and with the advent of more effective and comprehensive technologies, even more research will move to this culture independent, data driven approach. Further development in multi-omic databases will fuel research in improving the technologies related to each omics data set. Understanding the methods that bacteria use to interact with their environment through analysis of simultaneous omics data sets will result in the development of network links between the data sets themselves. Common links that are regularly observed between omics data sets will lead to greater molecular understanding and will be incorporated into traditional interaction networks. Extracting and depicting these complex interactions is an intrinsic issue associated with big data sets that multi-omics produce. Complex software pipelines play an important role in making sense of these data. To this end, we will briefly mention the more prominent software used for these integration processes.

## Data Integration and Computational Limitations

The integration of data sets generated in multi-omics research is by no means trivial. Each omics technology naturally consists of different types of data complicating the analysis to begin with. This is further complicated by the sheer volume of information that must be sifted through, particularly with meta-omics data. Software pipelines have been developed to manage this seemingly impossible task. Programs are developed to create models that can predict outcomes when multi-omics data is inputted. The outcomes analyzed are frequently disease states that can be described in terms of multi-omic data sets using these techniques. These pipelines generally fit into one of three approaches.

These models approach the issue with different methods, but all are valuable assets available to integrate the data. However, each of these approaches has limitations when negotiating these data. These limitations manifest in difficulty transforming differing data sets, combining massive input matrices and over fitting training data. Most pipelines adopt one of these approaches as a broad starting point. Researchers will have nuanced differences in how they treat types of data and how they weight connections between their data points.

**FIGURE 2:**
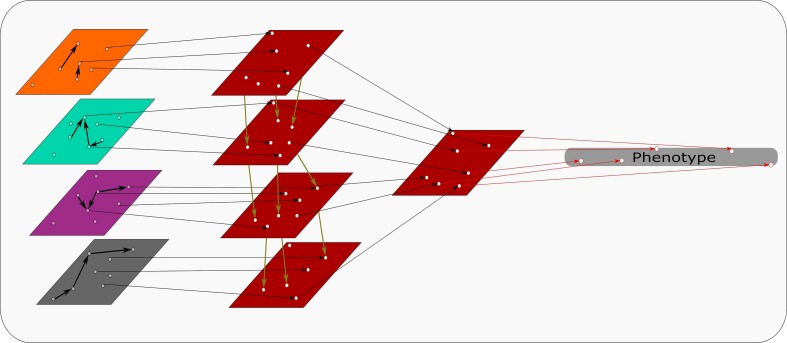
Transformation based integration: Each omics data set is transformed into comparable input matrices. Relevant identifiers are united to build the predictive model from all transformed data sets. This model discerns phenotypic traits that can be quantified using multi-omics data.

A creative use of a transformation based approach has led to remarkable results in liver cancer survival prediction (Chaudhary et al., 2018). This model developed by Chaudhary et al., 2018 uses a deep learning method autoencoder and a single variate cox-PH model to choose features associated with survival. K-mean clustering is applied to these features to determine survival-risk groups. The omics data sets are then ranked via an ANOVA and features common with the predicting set are chosen. This step is depicted in Figure 2, where omics data sets are transformed into survival-risk predicting sets. These ranks can then be compared and combined. The final survival-risk labels are generated from the top features chosen by this method (Chaudhary et al., 2018). By incorporating microRNA seq, RNA seq, methylation and genomic information this study highlights the potential powerful use for multi–omics data when appropriate software can realize its potential.

**FIGURE 3:**
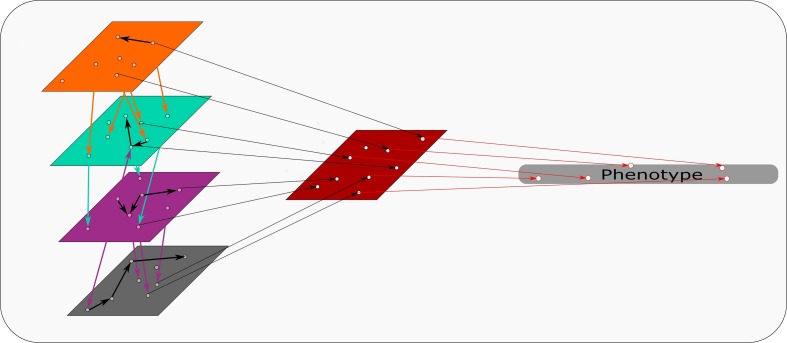
Concatenation based integration: Omics data sets (colored rectangles) are combined at the beginning of concatenation based integration. Identifiers are determined between and within each omics set. These identifiers are combined and used as a model to discern specific attributes within the phenotype.

**FIGURE 4:**
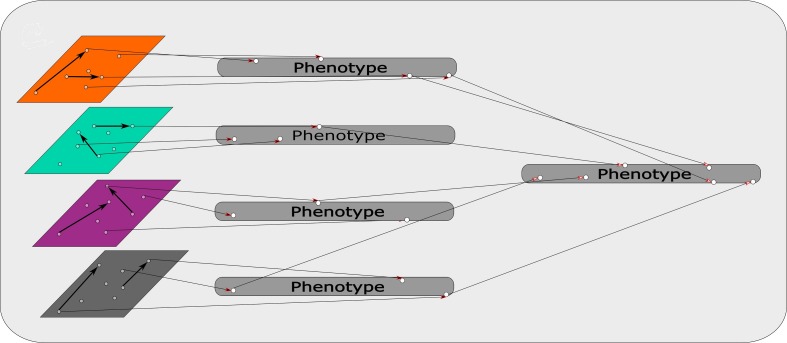
Model based integration: Each omics data set is used as a model to determine identifiers of traits within the phenotype. The phenotypic traits that are discerned from each omics data set/model are weighted based on their capacity to predict the phenotype. These weighted identifiers are then combined and ultimately predict the phenotype.

An R based software, mixOmics, presents a recent example of a modified concatenation-based approach (Rohart et al., 2017). This R based software uses its “Diablo” process for this approach. Diablo incorporates all the input variables into a single input matrix. During this process interactions and associations between data sets are factored into the single input matrix. Figure 3 represents a schematic of this approach, combining the different omics sets while taking interactions between data sets into account. This adds another level of complexity that suits multi-omics approaches specifically, creating a more powerful input matrix from which the model is derived. This software also contains another pipeline, namely “MINT”, which is a model-based integration where data sets of the same type, e.g., transcriptomics, from different studies are combined to produce a model. Each type of data set predicts aspects of the phenotype as illustrated in Figure 4. The predictions determined by each model are weighted based on their propensity to determine the phenotype. These input models are subsequently combined to generate a final prediction model (Rohart et al., 2017).

These exciting new software packages encompass each of the major methods of generating models for integrating omics data. Many developers are constantly updating and upgrading their platforms to incorporate the latest data sets and produce the most effective models (Medina et al., 2010; Alonso et al., 2015). Many concatenation based approaches have been developed to address nuanced different data sets (Fridley et al., 2012; Kim et al., 2013). Cancer survival prediction has seen significant success utilizing transformation and model based approaches. This progress has been observed using multiple genomic data sets to accurately assess ovarian cancer survival and in predicting instigators of melanoma from gene expression data and chromosomal copy number variation (Akavia et al., 2010; Kim et al., 2013).

More powerful models are constantly being developed, incorporating ever expanding data sets while integrating even more types of omics data. These advances are essential to keep up with the rapidly increasing volumes of data and to address the current limitations associated with many original data sets (Pinu et al., 2019). Several studies have emphasized the over estimation of significant results and several contradictory outcomes in multi-omics data sets in many high impact publications (Ioannidis, 2005; Ioannidis and Trikalinos, 2005). In these cases, genuine heterogeneity within samples in genome wide association studies is considered statistically important disease specific information (Ioannidis et al., 2003; Kavvoura et al., 2008). Software tools must understand and incorporate these issues, while avoiding the risk of false negatives due to too harshly correcting data. For more information on the challenges associated with integrating these data sets, the reader is directed to the following review articles (Palsson and Zengler, 2010; Gomez-Cabrero et al., 2014; Fondi and Lio, 2015). For the reasons detailed above, there must be an element of responsibility on biologists to negate some of these limitations by developing a workable level of understanding regarding the most suitable software model for their experimental questions.

## Conclusions

The mechanisms responsible for generating omics information have seen considerable progress in recent years. The advent of new technologies, such as third generation sequencing, is capable of transforming the level of information available to researchers. These advances have placed the integration of multiple omics data sets within reach of more scientists. This availability will result in substantial advances in all aspects of microbial work from delineating specific functions to understanding their role in complex ecosystems. This review assesses the advantages to a high dimensional systems level approach when analyzing organisms and systems simultaneously. The fortuitous position that LAB research finds itself in is also discussed. LAB are a group of microbes with a wealth of data already available in a variety of databases. Due to this position, research focused on this group is poised to take full advantage of the progress in multi-omics research. As the field of molecular biology becomes a data intensive one, it is critical that biologists keep up with this trend. Researchers must develop skills in data processing, develop an understanding of the mechanisms behind software they utilize and be flexible to incorporating new technologies into their workflows. This task is challenging, but the rewards available with multi-omics are substantial.

## Author Contributions

SO drafted the initial manuscript. CS and RR provided critique and corrections and all worked together in the construction of the final review.

## Conflict of Interest

The authors declare that the research was conducted in the absence of any commercial or financial relationships that could be construed as a potential conflict of interest.
